# A clinicopathological study of parotid carcinoma: 18-year review of 171 patients at a single institution

**DOI:** 10.1007/s10147-018-1266-7

**Published:** 2018-03-21

**Authors:** Akira Nishikado, Ryo Kawata, Shin-ichi Haginomori, Tetsuya Terada, Masaaki Higashino, Yoshitaka Kurisu, Yoshinobu Hirose

**Affiliations:** 1 Department of Otorhinolaryngology, Head and Neck Surgery, Osaka Medical College, 2-7 Daigaku-Machi, Takatsuki, Osaka 569-8686 Japan; 2 Department of Pathology, Osaka Medical College, Takatsuki, Osaka Japan

**Keywords:** Parotid carcinoma, Pathological grade, Treatment, Survival, Immunohistochemistry

## Abstract

**Background:**

This study investigated the clinical outcomes of patients with parotid carcinoma at a single institution during an 18-year period, with the focus on diagnosis, treatment, and survival.

**Methods:**

The subjects were 171 patients with parotid carcinoma treated at our department during the 18-year period from September 1999 to August 2017. There were 19 patients in stage I, 65 patients in stage II, 22 patients in stage III, and 65 patients in stage IV. The symptoms, preoperative diagnosis, node metastasis, survival rate, prognostic factors, and immunohistological findings were investigated.

**Results:**

Preoperative diagnosis of the histological grade by fine-needle aspiration cytology was only possible in 34% of the patients, while the histological grade was correctly determined by frozen section biopsy in 72%. The overall frequency of lymph node metastasis was 29%, with 59% in patients with high-grade carcinoma and only 6% in those with low-/intermediate-grade tumors. The disease-specific 5-year survival rate was 100% for patients in stage I, 95.2% in stage II, 70.4% in stage III, and 45.1% in stage IV. Multivariate analysis showed that the pathological grade was the most important prognostic factor. Immunohistological investigation showed patients with HER-2 or androgen receptor-positive tumors had a significantly worse prognosis.

**Conclusions:**

Although a high-grade tumor is the most important prognostic factor, preoperative diagnosis of the grade was not always accurate. Since advanced cancer has a poor prognosis with a limited response to surgery and radiation therapy, development of new treatment strategies, such as molecular-targeting therapies directed against HER-2 and AR, is required.

## Introduction

Parotid carcinoma is a rare tumor that accounts for approximately 0.5% of all carcinomas and represents less than 5% of head and neck cancers [1, 2]. While most oral, pharyngeal, and laryngeal cancers are squamous cell carcinomas, parotid carcinoma varies widely and is classified into 23 histopathological types according to the 2017 classification of the World Health Organization [3]. In addition, a single histopathological type often has several pathological grades. Therefore, treatment planning cannot be based on tumor staging alone, and preoperative diagnosis of both the histopathological type and pathological grade is also important. Although there have been various reports of methods for preoperative histopathological diagnosis and determination of the pathological grade, including fine-needle aspiration cytology (FNA), diagnosis depends largely on the pathologist’s experience and results can be inconsistent among different institutions [4, 5]. Surgery is the first-line therapy for parotid carcinoma, but the incision plan and the criteria for facial nerve preservation have not been standardized. In addition, the indications for postoperative radiation therapy are still under discussion [6, 7].

It is not easy to assess the prognostic factors for parotid carcinoma or the survival rate for several reasons, including the low incidence of this disease with various histopathological types and pathological grades [3], the high prevalence of low-grade carcinoma that needs long-term follow-up [8], and the difficulty in following a particular diagnosis and treatment plan for a long period. In particular, since treatment should be determined according to the pathological grade and histopathological type, accurate results can only be obtained from accumulated data on patients managed according to a consistent plan for diagnosis and treatment. Therefore, it is difficult to find solid evidence based on patient data obtained from multiple institutions.

During the past 18 years, our department has followed an essentially consistent diagnostic and treatment approach for the management of parotid carcinoma. Accordingly, we conducted a retrospective clinicopathological investigation of a total of 171 patients accumulated during that 18-year period.

## Patients and methods

### Patients

During the 18 years from September 1999 to August 2017, 171 new patients with parotid carcinoma were treated at our department. Classification of the parotid tumor from T1 to T4 showed that the number of patients in each T category was 21, 76, 23, and 51, respectively. Lymph node metastasis was diagnosed preoperatively in 44 patients. When the 171 parotid carcinoma patients were classified by stage, there were 19 patients in stage I, 65 patients in stage II, 22 patients in stage III, and 65 patients in stage IV. We used pathological TNM classification (Table 1). With respect to classification by pathological grade of low, intermediate, or high, the number of patients was 17, 81, and 73, respectively (Table 2). Among the 171 patients, survival data were obtained for 130 patients whose outcome could be confirmed. When these 130 patients were classified by pathological stage, there were 16 patients in stage I, 50 patients in stage II, 16 patients in stage III, and 48 patients in stage IV. When the tumor was classified by pathological grade as low/intermediate grade or high grade, the number of patients in each category was 80 and 50, respectively. The main histopathological types were mucoepidermoid carcinoma in 44 patients, carcinoma ex pleomorphic adenoma (CXPA) in 25 patients, adenoid cystic carcinoma in 21 patients, acinic cell carcinoma in 20 patients, and salivary duct carcinoma (SDC) in 13 patients (Table 3).

**Table 1:** TN classification of parotid carcinoma (*n* = 171)

	N0	N1	N2a	N2b	N2c	N3	Total
T1	19	2	0	0	0	0	21
T2	65	1	0	10	0	0	76
T3	17	2	0	4	0	0	23
T4	26	7	0	15	3	0	51
Total	127	12	0	29	3	0	171

**Table 2:** Relationship between histopathological grade and stage of parotid carcinoma (*n* = 171)

Stage	Pathological grade			Total
	Low	Intermediate	High	
I	4	11	4	19
II	10	46	9	65
III	0	14	8	22
IV	3	10	52	65
Total	17	81	73	171

**Table 3:** Histopathological types of parotid carcinoma (*n* = 171)

Histopathological Type	Cases (*n*)
Mucoepidermoid carcinoma	44
Carcinoma ex pleomorphic adenoma	25
Adenoid cystic carcinoma	21
Acinic cell carcinoma	20
Salivary duct carcinoma	13
Basal cell adenocarcinoma	10
Squamous cell carcinoma	9
Epithelial–myoepithelial carcinoma	8
Adenocarcinoma, not otherwise specified	6
Myoepithelial carcinoma	6
Others	9
Total	171

### Methods

#### Symptoms and signs

The chief malignant symptoms/signs reported in patients with parotid carcinoma, i.e., spontaneous pain/tenderness, adhesion to the surrounding tissues (tumor mobility), and facial palsy, were investigated in relation to the pathological grade and stage.

#### Preoperative diagnosis

FNA diagnosis was investigated in 163 patients in whom the final histology could be determined. Preoperative FNA was performed only once under US guidance, and the tumor was diagnosed by 2 pathologists (coauthors). Frozen section biopsy (FS) was performed in 117 patients and the lesion was also diagnosed by 2 pathologists (coauthors).

#### Cervical lymph node metastasis

Preoperative diagnosis of lymph node metastasis was performed with ultrasonography and contrast CT. In principle, total neck dissection (levels I–V) was done if metastasis was positive (N+) and the pathological grade was high, while elective neck dissection (END) (levels I–III and the upper part of level V) was performed for low-/intermediate-grade N0 tumors. Neck dissection was performed in 107 out of 171 patients, including total neck dissection in 40 cases and END in 67 cases. Of the 107 patients undergoing neck dissection, the metastatic sites were investigated in 41 patients who were histopathologically positive for lymph node metastasis (pN+).

#### Prognostic factors and survival rate

Univariate analysis and multivariate analysis were performed to investigate the prognostic factors in the 130 patients whose outcome could be confirmed. The factors employed in univariate analysis were age, gender, symptoms, T factor, N factor, stage, pathological grade, and presence/absence of radiation therapy. In addition, the survival rate was investigated in relation to stage and pathological grade. Survival rate was also investigated in the 103 patients in whom immunohistological examination was performed and the outcome could be confirmed. The follow-up period ranged from 4 months to 18 years.

#### Immunohistochemistry

Tumor tissue sections (4 µm thick, formalin-fixed, and paraffin embedded) were assessed by immunohistochemistry using the following primary antibodies: anti-HER2 (Nichirei, polyclonal), anti-AR (DAKO, AR441, monoclonal), and anti-EGFR (Nichirei, 31G7). Immunohistochemical studies were performed by a pathologist (Y. K.) according to the manufacturer’s instructions. Appropriate positive and negative controls were employed for all conditions.

Positivity for HER2, AR, or EGFR was scored from 0 to 3+ based on the percentage of positive tumor cells and the intensity of staining as follows: 0 (no staining or staining of < 10% of the tumor cells), 1+ (faint and partial staining of 10% of the tumor cells), 2+ (weak to moderate complete membrane staining of ≥ 10% of the tumor cells), and 3+ (strong complete membrane staining of ≥ 10% of the tumor cells [9, 10]). Scores of 2+ or 3+ for HER2 were classified as overexpression because it is widely recognized that only scores of 2 or 3 on immunohistochemical analysis are frequently associated with HER2 gene amplification [11, 12]. Based on the criteria for evaluating the response of colorectal carcinoma to anti-EGFR therapy, a score of 0 was considered to be negative for EGFR, and scores of 1+ to 3+ were considered to be positive. For AR, only a score of 3+ was classified as overexpression.

#### Treatment

Surgery was performed in 158 patients and 13 patients were inoperable. Local excision was performed according to the size and pathological grade of the tumor. Parotidectomy was done in 76 patients, while subtotal parotidectomy and total/extended parotidectomy were performed in 41 patients each. The facial nerve was preserved in 70 patients, partially excised in 36 patients, and completely excised in 52 patients. Operations with facial nerve preservation were performed in all nine cases which could not be diagnosed as malignant suspected or benign/inadequate by frozen section. Neck dissection was carried out in 107 patients, which was total neck dissection in 40 patients and END in 67 patients. Postoperative radiation therapy was given for high-grade tumors, positive lymph node metastasis, T4 disease, and a positive resection margin. Using these criteria, postoperative radiation therapy was performed in 73 patients, and the mean radiation dose was 60 Gy.

## Results

### Symptoms and signs

Malignant symptoms and signs were compared between 98 patients with tumors of low/intermediate pathological grade and 73 patients with high-grade tumors. Spontaneous pain/tenderness was noted in 40 patients (41%) from the low-/intermediate-grade group and 49 patients (67%) from the high-grade group. Adhesion to the surrounding tissues (restricted mobility or fixation) was observed in 44 patients (45%) from the low-/intermediate-grade group versus 69 patients (95%) from the high-grade group. Facial palsy was found in 5 patients (5%) from the low-/intermediate-grade group versus 27 patients (37%) from the high-grade group. The frequency of both symptoms and objective findings was significantly higher in the high-grade group (*p* < 0.001). We have no experience of preoperative facial palsy in 633 cases of benign parotid tumors.

Malignant symptoms/signs were also investigated by stage. Among the 19 patients in stage I, spontaneous pain/tenderness was observed in 10 patients (53%), and adhesion to the surrounding tissues was seen in 7 patients (37%); however, none of them had facial palsy (0%). These signs were, respectively, observed in 35, 48, and 0% of patients in stage II, 45, 68, and 0% of the patients in stage III, and 71, 92, and 49% of patients in stage IV. The frequency of both symptoms and objective findings was significantly higher in stage IV patients (*p* < 0.001) (Table 4).

**Table 4:** Signs and symptoms of parotid carcinoma-grade and stage

	Pain/tenderness	Mobility		Facial palsy
		Restricted	Fixed	
Grade				
Low/intermediate (98)	40	41	3	5
High (73)	49	41	28	27
Total (171)	89	82	31	32
Stage				
I (19)	10	7	0	–
II (65)	23	27	4	–
III (22)	10	14	1	–
IV (65)	46	34	26	32
Total (171)	89	82	31	32

### Preoperative diagnosis

**Fig. 1:**
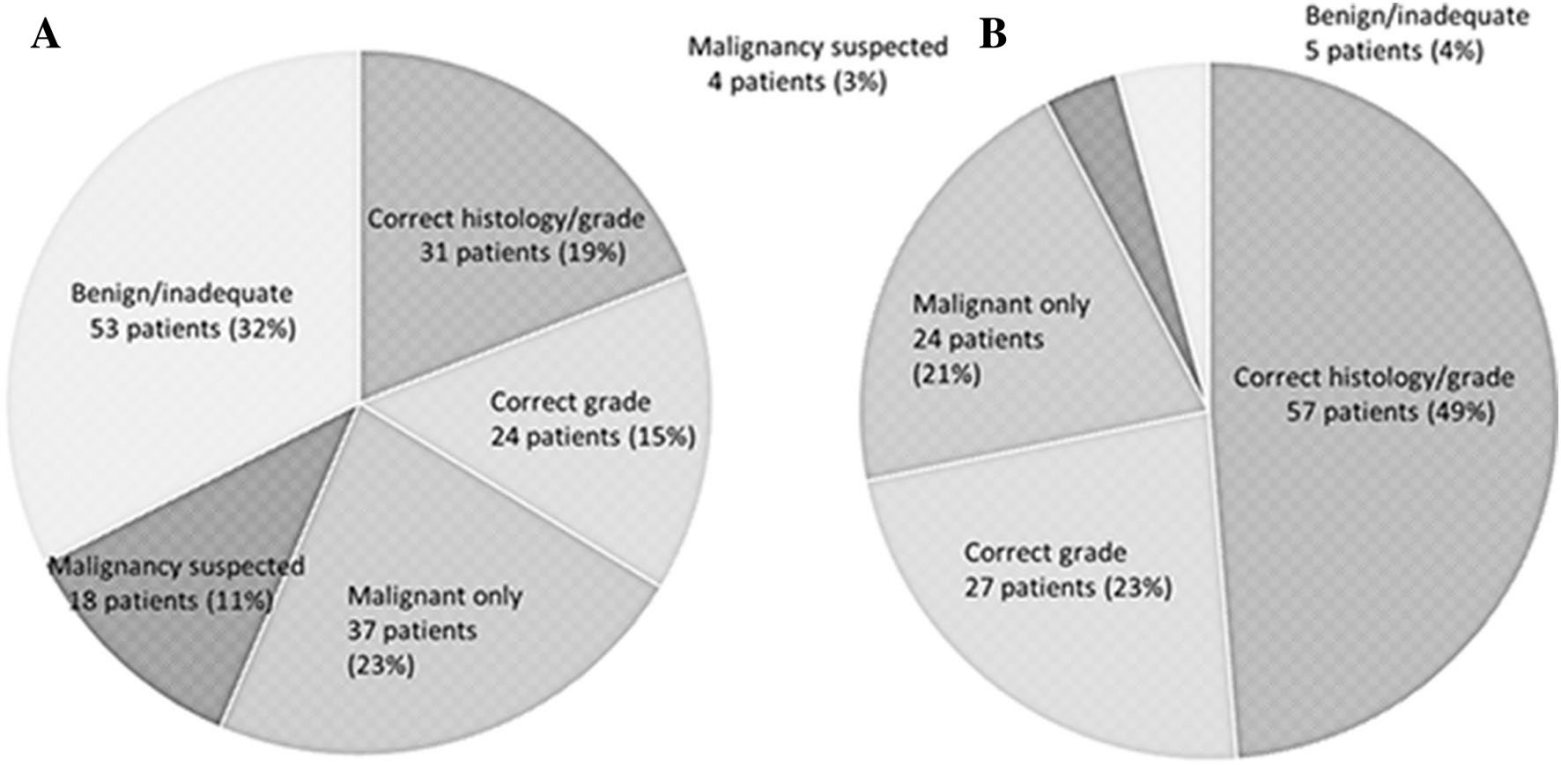
Results of fine-needle aspiration cytology (FNA) in 163 patients with parotid carcinoma (**a**). Only 31 patients (19%) were diagnosed correctly for both histological type and grade, and 53 patients (32%) with parotid carcinoma were diagnosed as having a benign tumor or inadequate material by FNA. Results of frozen section (FS) biopsy in 117 patients with parotid carcinoma (**b**). A total of 57 patients (49%) were diagnosed correctly for both histological type and grade by FS

The FNA diagnosis was evaluated in 163 patients whose histopathological type could be determined. Both the histopathological type and pathological grade were diagnosed correctly in 31 patients (19%), while the pathological grade was diagnosed correctly (histopathological type unknown) in 24 patients (15%). In addition, only malignancy was diagnosed (histopathological type and pathological grade unknown) in 37 patients (23%), malignancy was suspected in 18 patients (11%), and benign disease or an inadequate sample was found in 53 patients (32%) (Fig. 1a).

FS was performed in 117 patients and the results were compared with the final histopathological diagnosis. Both the histopathological type and pathological grade were diagnosed correctly in 57 patients (49%), while the pathological grade was diagnosed correctly (histopathological type unknown) in 27 patients (23%). Only malignancy was diagnosed (histopathological type and pathological grade unknown) in 24 patients (21%), malignancy was suspected in 4 patients (3%), and benign disease or an inadequate sample was found in 5 patients (4%) (Fig. 1b). Non-diagnostic cases by frozen section were predominant in basal cell carcinoma (7/10), epithelial–myoepithelial carcinoma (3/8), and carcinoma ex pleomorphic adenoma (5/25).

### Cervical lymph node metastasis

According to the preoperative diagnosis, the number of patients in the N0, N1, and N2 categories was 127, 12, and 32, respectively (Table 1). Neck dissection was performed in 69 of the 127 N0 patients, among whom 8 patients were diagnosed with pN+ and 61 patients with pN0. Neck dissection was performed in all 12 N1 patients, with 11 patients being diagnosed with pN+ and 1 patient with pN0. Neck dissection was performed in 26 out of 32 N2 patients, among whom 22 patients were diagnosed with pN+ and 4 patients with pN0. Although neck dissection was not performed in the remaining 6 inoperable patients, lymph node metastasis was confirmed by FNA in all cases. Among the N0 patients who did not undergo neck dissection, lymph node metastasis was observed in 2 cases. When all of these cases are totaled, lymph node metastasis was positive in 49 of the 171 patients (28.7%). When classified by pathological grade, 6 out of 98 patients in the low-/intermediate-grade group (6.1%) had metastasis, whereas 43 out of 73 patients in the high-grade group (58.9%) were positive for metastasis. When stratified by T classification, 2 out of 21 patients with T1 disease were positive for metastasis, as were 10 out of 74 patients with T2 disease, 9 out of 23 patients with T3 disease, and 28 out of 48 patients with T4 disease. The 41 patients with positive metastatic nodes after neck dissection were investigated to assess the sites involved. The largest number of patients had level II metastasis (30 patients, 74%), followed by metastasis to peri/infra parotid nodes in 25 patients (62%). Thus, metastasis was in the order of level III, IV, V, and I, with the frequency of metastasis being 45, 31, 17, and 12%, respectively (Table 5).

**Table 5:** Sites of lymph node metastasis in patients with parotid carcinoma

Level	No. of patients
I	5 (12%)
II	30 (74%)
III	19 (45%)
IV	13 (31%)
V	7 (17%)
Periparotid	25 (62%)

### Prognostic factors and survival

**Fig. 2:**
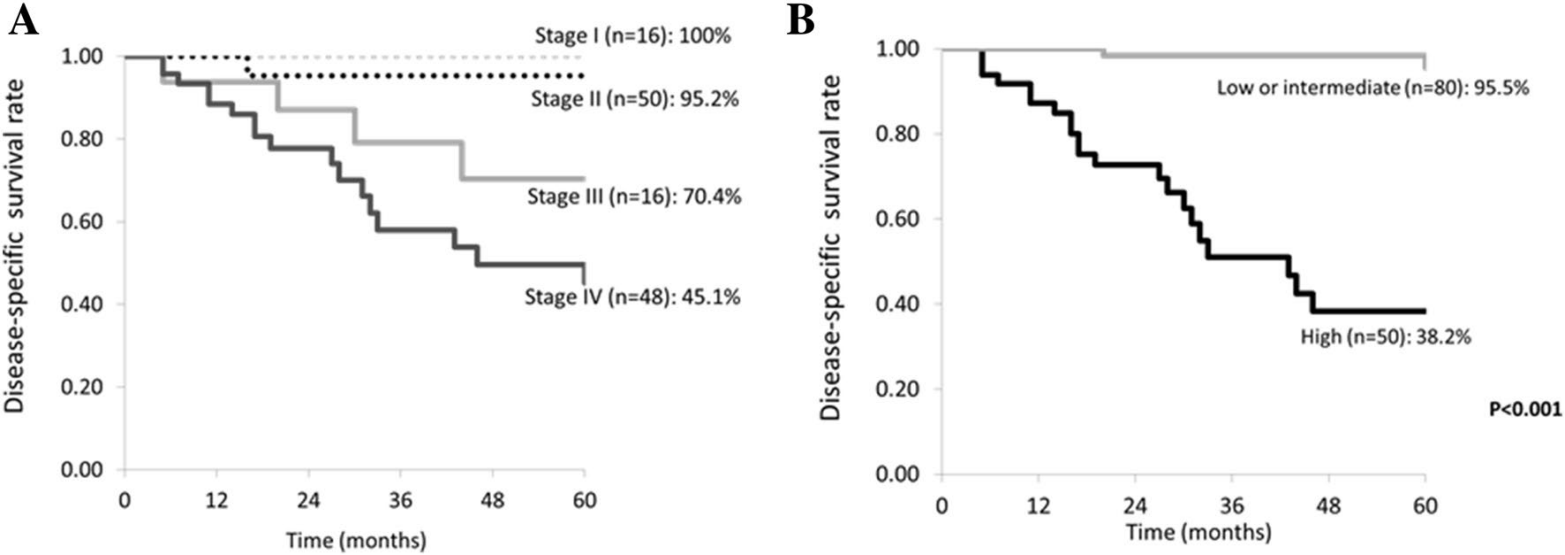
Disease-specific 5-year survival rate of 130 patients with parotid carcinoma. Survival is stratified by stage (**a**) and by histopathological grade (**b**)

Analysis of factors affecting the outcome showed that gender (male), adhesion to surrounding tissues, T factor (T4), N factor (N+), stage (IV), pathological grade (high grade), and radiation therapy (present) were strong prognostic indicators (*p* < 0.001). Multivariate analysis of the strong factors for poor prognosis extracted by univariate analysis was done using the Cox hazard model, revealing that high pathological grade was a significantly poor prognostic factor (Table 6). The overall disease-specific 5-year survival rate (DSS) was 75.1%, while the DSS for patients in stages I–IV was 100% (16 patients), 95.2% (50 patients), 70.4% (16 patients), and 45.1% (48 patients), respectively (Fig. 2a). The disease-free 5-year survival rate (DFS) for patients in stages I–IV was 100, 92.1, 64.0, and 44.3%, respectively. When DSS was stratified by pathological grade, it was 95.5% for the low-/intermediate-grade tumors (50 patients) and 38.2% for high-grade tumors (80 patients) (Fig. 2b). In addition, DFS stratified by pathological grade was 93.1% for the low-/intermediate-grade disease and 35.6% for high-grade disease. When DSS was stratified by the diagnosis of FS, there was no significant difference in the DSS among FS diagnosis. With focus on high-grade cases, the well-diagnosis group by FS had a poorer DSS than the non-diagnosis group, which was due to the high number of advanced stage in the well-diagnosis group.

**Table 6:** Univariate and multivariate analysis of factors influencing 5-year disease-free survival (DFS)

Factors		Patients (%)	5Y-DFS	*p* value
Age	< 60 years old	65 (50)	85.6	0.03
	≥ 60 years old	65 (50)	59.8	
Sex	Male	73 (56.2)	56.5	< 0.001
	Female	57 (43.8)	96.1	
Preoperative				
Pain/tenderness	+	65 (50)	65.4	0.02
	−	65 (50)	82.3	
Adhesion	+	85 (65.4)	60.2	< 0.001
	−	45 (34.6)	97.1	
Facial palsy	+	26 (20)	46.4	0.02
	−	104 (80)	80.6	
T stage	T1–T3	89 (68.5)	86.6	< 0.001
	T4	41 (31.5)	43.5	
N stage	N0	98 (75.4)	85.1	< 0.001
	N1–3	32 (24.6)	39.3	
Stage	I–III	82 (63.1)	89.9	< 0.001
	IV	48 (36.9)	44.2	
Pathological grade	Low/intermediate	80 (61.5)	95.1	< 0.001
	High	50 (38.5)	36.6	
Radiation therapy	+	58 (44.6)	53.0	< 0.001
	−	72 (55.4)	90.4	

	Factors	DFS		
		HR (95% CI)	*p* value	
	Low/intermediate or high	0.0887 (0.0179–0.4396)	0.003

### Immunohistochemistry

**Fig. 3:**
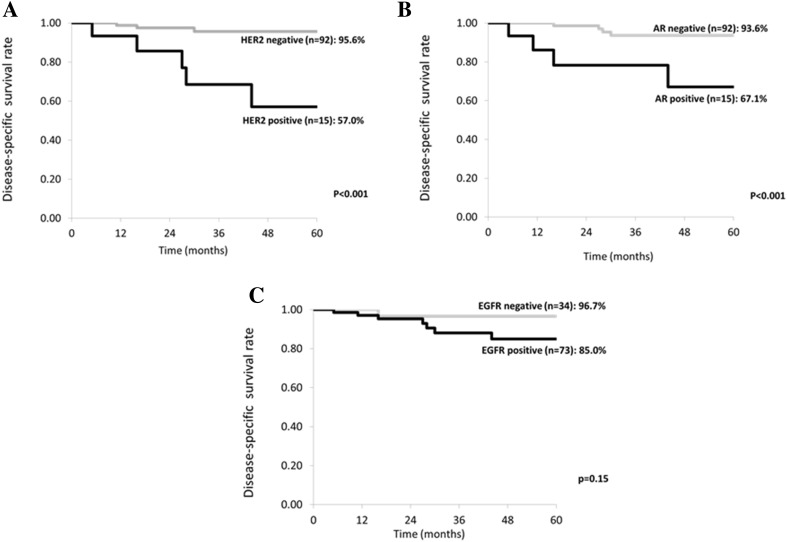
Disease-specific 5-year survival rate (DSS) stratified by immunohistochemical findings of HER2 (**a**), AR (**b**) and EGFR (**c**) in 107 patients. DSS was significantly worse in HER2 or AR positive patients than in HER2 or AR negative patients. However, there was no significant difference of DSS in relation to EGFR expression

Immunohistochemistry was investigated in 107 of the 171 patients whose outcome was confirmed after immunostaining (Fig. 3a–c). Among them, 15 patients were HER2 positive and 92 patients were HER2 negative. DSS was 57.0% in the HER2-positive patients and 95.6% in the HER2-negative patients, while DFS was 33.0 and 87.0%, respectively. HER2-positive patients had a significantly worse prognosis (*p* < 0.001). There were 15 AR-positive patients and 92 AR-negative patients. DSS and DFS of the AR-positive patients were 67.1 and 55.0%, respectively, whereas DSS and DFS of the AR-negative patients were 93.6 and 82.4%, respectively, with the prognosis of AR-positive patients being significantly worse (*p* < 0.001). There were 73 EGFR-positive patients and 34 EGFR-negative patients. DSS and DFS of the EGFR-positive patients were 85.0 and 76.7%, respectively, whereas DSS and DFS of the EGFR-negative patients were 96.7 and 81.9%, respectively, showing no significant difference (*p* = 0.15).

When the 15 HER2-positive patients were investigated histopathologically, 5 patients had SDC, 6 had CXPA carcinoma ex pleomorphic adenoma, and 4 patients had squamous cell carcinoma, mucoepidermoid carcinoma, basal cell carcinoma, or adenocarcinoma (NOS). With regard to pathological grade, 13 patients had high-grade tumors, and the number of patients in stages I–IV was 2, 3, 2, and 8, respectively. All of the patients with HER2-positive tumors were men.

## Discussion

It has been reported that parotid carcinoma is infrequent and has an incidence of 1.3 per 100,000 persons in the general population [13]. In addition, parotid carcinoma has various pathological grades and histopathological types, thus making it difficult to accumulate and investigate a large series of patients with the disease.

Pathological grade is one of the most important prognostic factors for parotid carcinoma. The correct preoperative diagnosis is necessary for adequate surgical management, so pretreatment diagnosis of the pathological grade is important for deciding the treatment plan. Although the pathological grade can be diagnosed by FNA and FS, the results are not always satisfactory and diagnosis largely depends on the experience of the pathologist. Thus, diagnostic expertise for parotid carcinoma differs among institutions and results in different treatment plans. During the past 18 years, our department has followed a consistent diagnostic and treatment policy. For the reasons mentioned above, we consider that it is significant to investigate a large case series of parotid carcinoma managed at a single center.

Pain is the most important feature that cannot be overlooked among the symptoms of parotid carcinoma. In this series, pain was noted in 52% of all parotid carcinoma patients. Pohar et al. [14] reported that pain was a symptom in 34% of their patients, while Godballe et al. [15] reported pain in 31% of parotid carcinoma patients and stated that pain was a poor prognostic factor. While Stodulski et al. [16] reported that pain was present in 40% of parotid carcinoma patients, they also concluded that it was not a prognostic factor. In this study, classification by pathological grade showed that pain was present in 67% of patients with high-grade disease and even 41% of those with low/intermediate disease. Particularly in patients with low-/intermediate-grade carcinoma, pain should not be overlooked because these tumors cause few other symptoms and often follow a course similar to benign lesions. It has generally been reported that benign tumors do not cause pain, and pain was only noted by 33 out of 668 patients (5%) with benign parotid tumors at our department. Since the frequency of pain was approximately 10 times higher in patients with parotid carcinoma than in those with benign tumors, the symptom of pain is considered to be the first indicator of possible malignancy. Although adhesion to the surrounding tissues is a slightly vague sign due to difficulty in performing quantitative assessment, it is an important feature of malignancy. It was reported that adhesion to the skin was observed in 9% of patients and adhesion to deep tissues in 13–18% of patients [15, 17, 18]. We consider that these findings correspond to “fixation” in our study, and the frequency was similar. It has been reported that the frequency of facial palsy is 9–25%, and a similar frequency of 20% was obtained in our study [17–19]. Facial palsy is known to be associated with recurrence and a poor prognosis [20, 21], and there was a significant difference of this sign between low-grade and high-grade tumors in the present study.

FNA is the only method for preoperative determination of the histological type and pathological grade, and it has been reported that the diagnostic accuracy of FNA for parotid carcinoma is unfavorable [22–24]. Although it is desirable to determine both the histological type and pathological grade, we consider that the latter is more important for treatment planning. Out of 163 patients, both the histological type and pathological grade were diagnosed correctly in only 19%, while the pathological grade was diagnosed correctly (histological type unknown) in 15%. Thus, the pathological grade was determined in 34% of the patients, i.e., only 1 out of 3. The diagnosis rate was comparatively favorable in patients with high-grade carcinoma, whereas it was unsatisfactory in patients with low-/intermediate-grade carcinoma [25]. The pathological grade was diagnosed by FS in 72% of the patients and, although this diagnosis rate is considerably higher compared with FNA, the problems to be noted here are that the diagnosis rate was worse for low-grade carcinoma as with FNA [25] and that surgery cannot be explained to patients preoperatively if the pathological grade is only diagnosed correctly by FS after the operation has already started. In histopathological types, non-diagnostic cases were predominant in basal cell carcinoma and epithelial–myoepithelial carcinoma. The correct diagnosis in FS, as well as FNA, was often difficult because these histopathological types have poor cellular atypia. We tried to study the relationship between prognosis and preservation/sacrifice of the facial nerve. However, it was impossible to analyze it because the number of cases was extremely imbalanced. In low-/intermediate-grade cases, only five cases were dead/recurrent among the total 75 cases. On the other hand, in high-grade cases, only four cases were preservation of the facial nerve among the 42 cases. These results suggest that it was impossible to analyze the relationship between prognosis and the facial nerve preservation/sacrifice, even though it was a very important and interesting point. As a result of multivariate analysis, we found that histological grade was a major prognostic factor and that correct preoperative diagnosis of pathological grade contributed to improvement of the surgical planning.

With regard to handling of lymph node metastasis in patients with parotid carcinoma, although most surgeons have no objection to neck dissection for N+ patients, consensus has not been reached on the indications for END in N0 patients [26]. Preoperative diagnosis of lymph node metastasis has limitations, and it is not rare for lymph node metastasis to be found by END (occult metastasis). Armstrong et al. [27] reported that occult metastasis was observed in 38% of patients. It was also found in 20% of patients by Zbaren et al. [28] and in 18% of the patients by Klussmann et al. [29]. In the present study, END was performed in 67 out of 127 patients clinically diagnosed as N0 (cN0), and pathological N+ (pN+) was observed in 8 patients. Among the 58 patients in whom END was planned, secondary lymph node metastasis was found in 2 cases. Accordingly, occult metastasis was detected in a total of 10 patients (8%). We consider that the occult metastasis rate is decreasing with the progress of imaging methods, but occult metastasis may exist in approximately 10% of patients. There have been various reports on the indications for END in patients with parotid carcinoma, and Sinha et al. [30] reported that they did not perform END at all. In contrast, Eneroth et al. [31] conducted END in all patients except for those with low-grade tumors. In addition, Armstrong et al. [27] reported that END should be conducted in patients with T3 or higher disease and patients with high-grade tumors. In the present study, lymph node metastasis was observed in 49 out of 171 patients (29%), and the lymph node metastasis rates were 59% for high-grade disease and 6% for low-/intermediate-grade disease. Based on these results, it may not be necessary to perform END in patients with low-/intermediate-grade tumors. However, it is still a problem that preoperative diagnosis of pathological grade by FNA and FS is not adequate. Even though not conducting END for low-/intermediate-grade tumors seems reasonable, this treatment policy should be based on preoperative diagnosis of the pathological grade. We define the range of END as levels I–III and the upper part of level V. We also preserve the sternocleidomastoid muscle, accessory nerves, and internal jugular vein. Considering that preoperative diagnosis of the pathological grade is difficult and there are limitations on the diagnosis of lymph node metastasis, we conclude that it is appropriate to perform END for all patients with parotid carcinoma since END causes less surgical stress. However, in the present study, among the 41 pN+ patients undergoing neck dissection, positive lymph nodes were found at level II in 74% and at the periparotid region in 62%. Since level II and the periparotid region can be approached by an S-shaped skin incision, another option is for FS of the lymph nodes to be performed first and neck dissection omitted if metastasis proves to be negative.

Salivary gland cancers are histologically similar to certain types of breast cancer [32]. Use of anti-HER2 therapy is currently approved for HER2 overexpressing breast cancer [33], which has led to interest in studying anti-HER2 treatment for salivary gland cancers. According to previous reports, the HER2-positive rate of salivary gland carcinoma is not always high. Glisson et al. [34] screened 137 patients and reported that the HER2-positive rate was 17%. In this study, there were 15 HER2-positive patients out of 107 patients (14%), which was a similar result. Among salivary gland carcinomas, the HER2-positive rate is reported to be higher for SDC [34, 35]. In our study, 5 out of 13 patients with SDC were HER2 positive. The HER2-positive rate is also higher in patients who have CXPA [36], and the positive rate is reported to be higher for invasive tumors. In this study, 6 out of 25 patients with CXPA were HER2 positive, and all 6 patients had invasive tumors with histological predominance of the SDC component. Since the number of HER2-positive patients is higher among those with invasive SDC and CXPA, it was reported that HER2-positive status is associated with a poor prognosis due to rapid progression. In this study, the DSS of HER2-positive patients was significantly worse. It has frequently been reported that the AR-positive rate is higher compared with that of HER2, and Locati et al. [37] found an AR-positive rate of 43%. In this study, the AR-positive rate was only 14% and was lower than in other reports. Similar to HER2, there were more AR-positive patients in the high-grade group, and 12 out of 15 AR-positive patients had high-grade tumors. With regard to the histopathological diagnosis, 5 patients were classified as SDC, while 6 patients had CXPA (3 high-grade tumors) and 3 patients had mucoepidermoid carcinoma (all high-grade tumors). The prognosis of AR-positive patients was poor, similar to that of HER2-positive patients. Since recurrence is frequent in patients with HER2- or AR-positive tumors, it is possible that molecular-targeting therapy is indicated for such disease. Recently, Keller et al. [38] summarized new concepts for personalized therapy in salivary gland carcinoma. According to this paper, there has not been any large-scale study on anti-HER2 and anti-AR therapies. However, we could find some studies stating that anti-HER2 and anti-AR therapies were effective in salivary gland carcinoma in small-scale studies. There have been a few reports showing that trastuzumab (an anti-HER2 antibody) is not effective as a monotherapy, but has some efficacy in combination with other agents [39, 40]. As antiandrogen therapy, Jasper et al. [41] reported that administration of bicalutamide was effective for AR-positive SDC. Since distant metastasis is frequently seen in patients with high-grade tumors, development of effective molecular-targeted therapy is needed.

## Conclusions

This is the largest published series of parotid cancer patients from a single center in Japan. Pathological grade was the most important prognostic factor according to multivariate analysis, suggesting that treatment planning for parotid cancer should depend on the pathological grade. However, preoperative diagnosis of the pathological grade by FNA was often incorrect or impossible. The disease-specific 5-year survival rate of patients in stages I to IV was 100, 95.2, 70.4, and 45.1%, respectively. Overexpression of HER2 and AR was observed in high-grade tumors, especially SDC, and was associated with a poor prognosis.
